# Antimicrobial and wound healing properties of a bacterial cellulose based material containing *B. subtilis* cells

**DOI:** 10.1016/j.heliyon.2019.e02592

**Published:** 2019-10-10

**Authors:** I.S. Savitskaya, D.H. Shokatayeva, A.S. Kistaubayeva, L.V. Ignatova, I.E. Digel

**Affiliations:** a Al-Farabi Kazakh National University, Al-Farabi av. 71, Almaty, 050038, Kazakhstan; b Aachen University of Applied Sciences, Heinrich-Mußmann-Street 1, Jülich, 52428, Germany

**Keywords:** Microbiology, Biotechnology, Microbial biotechnology, Bacterial cellulose, Wound healing, *Bacillus subtilis*, Biocomposite

## Abstract

A biocomposite composed of bacterial cellulose (BC) gel-film and *Bacillus subtilis* (BS) cells was obtained and characterized with a view to future biomedical applications. The inclusion of functional ingredient (10^10^/g viable BS cells) in the composite was carried out by their joint aggregation with the BC gel-film. Immobilized BS cells displayed high antagonistic activity towards causative agents of wound infections such as *Staphylococcus aureus, Staphylococcus epidermidis*, *Escherichia coli, Pseudomonas aeruginosa*. Application of the BC/BS-biocomposite for the treatment of excision wounds, performed on laboratory animals, stimulated reparative processes and shortened the healing time. Possible mechanisms of the wound-healing effect of BC/BS gel films are discussed. In this work we claim that the developed BC/BS-material can be positioned as a universal wound coating and sanitary-hygienic product.

## 1 Introduction

Development and application of many interesting wound dressing variants based on natural polymers was described in numerous scientific and patent reports of recent years [1]. A promising research direction is design of wound coatings based on the bacterial cellulose (BC) synthesized by *Gluconoacetobacter xylinus* [2, 3]. Such materials are biocompatible, i.e. not toxic, do not cause allergies and physical rejection [4]. In addition, they maintain the optimum moisture balance, can control wound exudates, stimulate healing, perfectly pass fluids and gases, can be painlessly applied and removed, absorb the products of tissue decay and serve as an almost insurmountable physical barrier to infection [5]. BC gel film has been used as a component wound coatings for skin transplantation as well as for treatment of various wounds and sores [6, 7, 8, 9]. Successful use of BC as a medical product was discussed in several publications and reports. The Biofills product was used for several skin injury treatments such as basal cell carcinoma/skin graft, severe body burns, facial peeling, sutures, dermabrasions, skin lesions, chronic ulcers, and both donor and receptor sites in skin grafts [10]. Another product called Xcell has an ability to simultaneously donate and absorb moisture from the wound, conforming to wounded and intact skin differently [11].

Despite the fact that BC gel-film is biocompatible to human body, it predominantly serves as a mechanical barrier protecting the wound surface from rapid drying and infection. To satisfy further requirements as a wound healing material, it has to be modified via improvement of its antibacterial activity. Reduction of infection risks is of general demand for most biomedical applications. Although BC does not possess an intrinsic antimicrobial property, due to its high water holding capacity and porosity it can absorb and slowly release an antimicrobial solution. Thereby, manufacture of antimicrobial wound coverings based on BC usually includes impregnation of antibiotics, biological and synthetic polymers with antimicrobial activity as well as inorganic antiseptics [12, 13]. However, it is well known that many causative agents of wound infections have multiple resistance to antibiotics. The resistance to antiseptics does not occur, however they only possess antimicrobial activity, without wound healing properties. In this regard, the wound dressing consisting of an agent which has both antimicrobial and tissue regeneration properties, would be very advantageous. Bacteria of *Bacillus* genus may serve as such an agent [14, 15]*.* These probiotic bacteria are well known for their production of a broad spectrum of antibiotics [16, 17] and bacteriocins [18]. The biosurfactant lipopeptides produced by these bacteria have antioxidant activity, which positively contribute to wound healing [19, 20]. Furthermore, proteolytic enzymes synthesized by *Bacillus* positively influence regeneration of tissues due to their thrombolytic effect, prevention of scar formation and necrotic tissues lysis [21, 22].

The positive effect of living probiotic bacteria of the *Bacillus* genus on the processes of wound healing устаноϑлен ϑ ряде исследоϑаний [23, 24, 25]. There are some pharmacological preparations in Russia for treatment of uninfected wounds, purulent-necrotic processes, burns and dermatitis: Bioseptin, Bactisporin, Sporobacterin, Bactisporinlast [26, 27]. However, in all cases, wounds are treated with either a suspension of *Bacillus subtilis* cells, or ointments containing the spores of *B. subtilis* and *B. licheniformis*.

The fibrillar reticulate structure of microbially manufactured BC is beyond what can currently be achieved artificially, and it looks like natural collagen in terms of its structure and morphology, which is attractive for cell immobilization and because it mimics extracellular matrix support [28]. The structural features of BC (micro- and nanofibrillar structure, high porosity and crystallinity) imply a huge potential for the creation of various composite materials on its basis [29]. Microfibril units occupy insignificant volume of the BC gel-film, therefore allowing introduction of various yeast and bacteria [30, 31, 32, 33, 34, 35, 36, 37, 38].

To the best of our knowledge, the potential use of bacterial cellulose-based wound healing material possessing advantageous properties due to the inclusion *B. subtilis* (BS) cells have never been explored before. The abovementioned fascinating features of both BC and BS motivated our research group for an attempt to synergistically combine them in a biocomposite. The aim of this work was design, characterization and examination of a BC-based wound healing material possessing advantageous properties due to the inclusion BS cells.

The performed tasks included:

1. Establishing of a reliable protocol for production of bacterial cellulose gel film and its modification with BS cells;
2. Characterization of (micro) structural, antimicrobial and proteolytic properties of the obtained BC/BS biocomposite;
3. *In vivo* studies on wound healing efficiency of the BC/BS biocomposite.

## 2 Materials and methods

### 2.1 Microbial strains

*Komagateibacter xylinus* C-3 and *Bacillus subtilis* P-2 strains were isolated at the Biotechnology Department, Al-Farabi Kazakh National University; the cultures were deposited in Republic Collection of Microorganisms (Astana, Kazakhstan); Gen Bank accession numbers are KU598766 and KY780502, respectively.

*Esherichia coli* ATCC 8799, *Pseudomonas aeruginosa* ATCC 9027, *Staphylococcus aureus* ATCC 6538, *Staphylococcus epidermidis* ATCC 14990 test cultures were obtained from American type culture collection.

### 2.2 Production and preparation of BC films

The production of BC films by *Komagateibacter xylinus* C-3was carried out using modified Hestrin-Shramm medium with the addition of 0.5% ethanol and 0.1% beer wort. Cultivation was carried out at 29–30 °C for 6–7 days. The developed gel-like cellulose pellicle was first purified by washing with deionized water for 5–7 min. Then it was treated with 1% (w/v) NaOH at 35 °C for 24 h to remove bacterial cells and the obtained acellular matrix was rinsed with deionized water until the pH of the rinsing solution was 6.8–7.2. BC films were sterilized by autoclaving.

### 2.3 *BS* immobilization in BC by «adsorption- incubation» procedure

In the «adsorption step», bacterial biomass (48-hour culture) at initial concentration of 10^10^ colony forming units (CFU) per ml was suspended in a phosphate buffer solution. Then BC pieces having an appropriate size (5сm^2^) were added to the cell suspension and incubated for 96 h upon continuous mild agitation. Finally, the liquid was decanted and the immobilized biocatalyst was washed with sterile water.

In the «incubation step», BC pieces containing bacterial cells were incubated in a sterile empty Erlenmeyer flasks with nutrient broth media at 30 °C for 96 h.

After incubation, the BC films with bacterial cells were washed in deionized water and digested by incubation with cellulase (100 mL/1 mL 0.05 M citrate buffer, pH 4.8, Sigma Aldrich). The bacterial cell suspensions obtained after digestion with cellulase were washed 3 times with 10 mL PBS and suspended in 1 mL PBS. The number of immobilized microorganisms was determined by two methods: (a) by spectrophotometric readings of the optical density (OD) of microorganism suspension at 650 nm (Infinite 200 PRO NanoQuant, Tecan, Switzerland) and (b) by performing quantitative plating on Nutrient agar (Biocorp). After incubation for 24 h at 37 °C, the grown colonies were counted and the number of CFU per 1 g of cellulose was determined. The OD of bacterial cultures indirectly reflects the number of viable and dead bacteria, whereas quantitative plating on bacteriological media determines only viable microbial cells.

Immobilization efficiency of bacterial cells in BC pieces was quantified by a method described by Leboffe and Pierce [39]. The average cell density (cells/g) was calculated as the number immobilized cells inside plus the number of immobilized cell outside all divided by the weight of the finished product. The immobilization efficiency H, % = total of immobilized cells onto BC/total inoculum cells∗100%. Obtained samples of BC/BS biocomposite were used in further work.

### 2.4 Scanning electron microscopy (SEM) studies of BC gel-films

The surface morphology of BC and BC/BS samples was observed by field emission scanning electron microscope JSM-7800F (Jeol, Japan). Prior to the SEM observation, the films were sputter coated with a platinum-palladium alloy (Pt/Pd 80/20). To characterize the microfibrillar structure of BC, mean value and standard deviation from 100 measurements were calculated.

### 2.5 Determination of antagonistic activity BC/BS biocomposite

Antimicrobial activity of BC/BS biocomposite was studied against Gram-negative *Escherichia coli ATCC 8799, Pseudomonas aeruginosa ATCC 9027* and Gram-positive *Staphylococcus aureus ATCC 6538* and *Staphylococcus epidermidis ATCC 14990* bacteria test cultures. The antimicrobial assessment was carried out by the following two methods:

#### 2.5.1 «Agar diffusion» method

The sterile Müller-Hinton nutrient medium was poured into sterile Petri dishes with a 4 mm thick layer. The plates were left at room temperature to solidify. Then a suspension of the test microorganisms (inoculum) was prepared. For that purpose a pure daily culture grown on a solid nutrient medium was used. Identical, clearly isolated colonies were selected. The loopful of the cells from a single colony was transferred to a test tube with sterile saline solution and the inoculum turbidity was adjusted to a McFarland standard 0.5, which corresponds to 1.5 × 10^8^ CFU (colony forming units) in 1 ml. The 2 ml inoculum pipetted on the surface of nutrient medium in a Petri dish was evenly spread over the surface of agar by shaking, then the excess liquid was removed. The opened cups were left at a room temperature for 10 min for drying. BC/BS films were placed on the surface of the nutrient medium. After the application of the biocomposite, the Petri dishes were incubated at 37 °C for 24 h, then the growth inhibition zone of the target microorganism was measured.

#### 2.5.2 The time-kill test

For bacteria, this test has been well standardized and described in M26-A document of CLSI [40]. It is performed in broth culture medium using three tubes containing a bacterial suspension of 5 × 10^5^ CFU/mL. BC/BS biocomposite in experimental series were placed in tubes inoculated with target microorganisms. The first tube contain the BC/BS biocomposite of 1 g weight and the second one is considered as the growth control. The incubation is done under suitable conditions for varied time intervals (0, 1, 6, 10 and 24h). Then, the percentage of dead cells is calculated relatively to the growth control by determining the number of living cells (CFU/mL) of each tube using the agar plate count method. The reduction ratio of the bacteria was evaluated by the following equation: R (%): A-B/A х 100% where R is the percentage reduction ratio, A is the number of bacterial colonies from the untreated bacteria suspension (without addition of BC/BS) and B is the number of bacterial colonies from the bacteria culture grown with BC/BS biocomposite. Generally, the bactericidal effect is obtained with a lethality percentage of 90% for 6h, which is equivalent to 99.9% of lethality for 24h [41].

### 2.6 Studying the proteolytic activity of BC/BS biocomposite

Protease enzyme detection of biocomposite was done using Luria casein agar [42]. Casein was autoclaved separately and after cooling was mixed aseptically in the medium before pouring of medium in plates. Pure BC and BC containing BS cells were placed on the surface of the Luria casein agar. The milk-casein digestion zone around the films containing BS were observed.

### 2.7 Wound healing studies

#### 2.7.1 Animals

The study was carried out on male mongrel rats weighing 180–200 g. The animals were kept on a standard ration of vivarium. The animal study was carried out abiding by the national regulations related to the conduct of experimentation. The experiments were guided by the “Rules on work with experimental animals” [43]. Three groups of male rats, five in each received the following treatment schedule: I - was covered with BC/BS biocomposite; II - was closed with a BC film; III - was served as the control (just cleaning the wounds with a physiologic serum). After the wounding process, each rat was housed in a sterilized cage and given autoclaved food and redistilled water in order to prevent bacterial infection.

The study was examined and approved by the Medical Faculty Ethics Committee of the al-Farabi Kazakh National University IRB00010790 (Protokol #IRB-A023).

#### 2.7.2 Induction of wounds

The wound healing activity of BC/BS biocomposite was evaluated on excision wound rats models. After total anesthesia with ketamine (100 mg/kg body weight) by intramuscular injection, a 2.0 cm longitudinal full thickness incision was made in the back of each rat. An excision wound margin was traced after wound creation by using graph paper and size was calculated. Wound contraction was evaluated on 3, 5, 7, 14 and 21 days until complete wound healing and expressed in percentage of healed wound size. The wounding day was considered as day 0. Wound closure, considered as percentage reduction of original wound size, was determined using the following formula:

$$\text{Wound ​closure},\text{ ​}\%=\frac{{\text{A}}_{0}\;–\;{\text{A}}_{\text{d}}}{{\text{A}}_{0}\;}\times 100,$$

where A_0_ was the initial wound size (day 0) and Ad was the wound size on present day (d). The period of epithelization was estimated as the number of days required for falling of the dead tissue without any residual raw wound. Digital photographs of the wounds were taken on days 1, 3, 7, 14 and 21.

### 2.8 Statistical analysis

Statistical comparison was performed using unpaired t-test and one-way analysis of variance (ANOVA) followed by Dunnett's test for multiple comparisons. All statistical analyses were performed using SPSS 16.0 software package (SPSS Inc., USA).

## 3 Results and discussion

### 3.1 Immobilization of *Bacillus subtilis* cells in BC by «adsorption-incubation» method for maximizing the cell number in BC

**Fig. 1:**
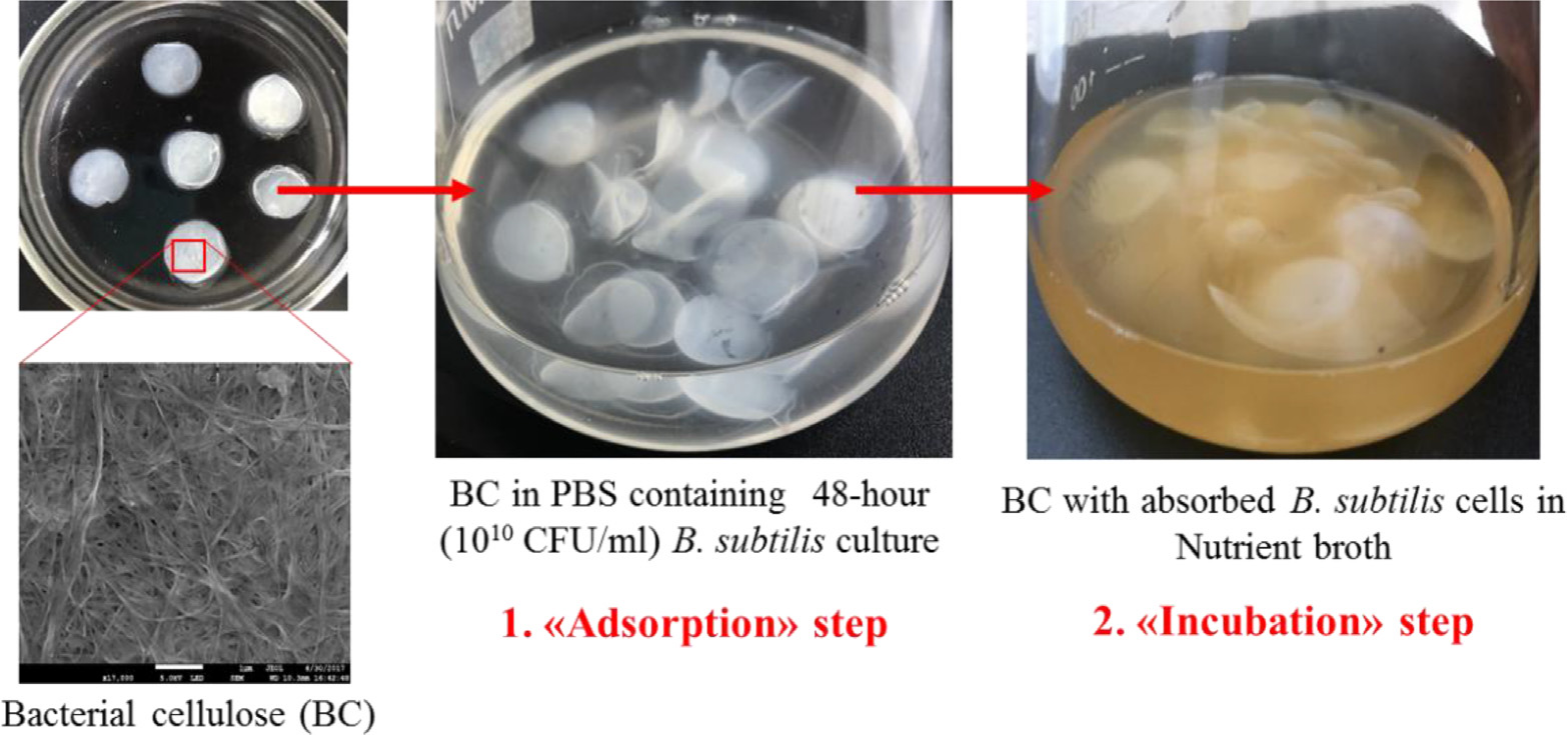
Experimental design of bacterial immobilization BS/BS films by «adsorption-incubation» method.

The strain BS Р-2 displaying high levels of antimicrobial and proteolytic activity was used as a «functional agent» [44]. For its immobilization an «adsorption-incubation» method, developed by Nguyen D.N. with coauthors was used [45]. The scheme of the experiment is shown in Fig. 1.

As BC was soaked in the cell suspension during the “adsorption” step, it became inflatable and the bacteria were adsorbed onto the BC surface due to its porous structure and properties In the incubation step, the immobilized cells' growth was supported by the nutritional compounds that diffused into BC's space. In this step, the density of immobilized bacterial cells not only increased significantly, but was also maintained for 96 h.

**Fig. 2:**
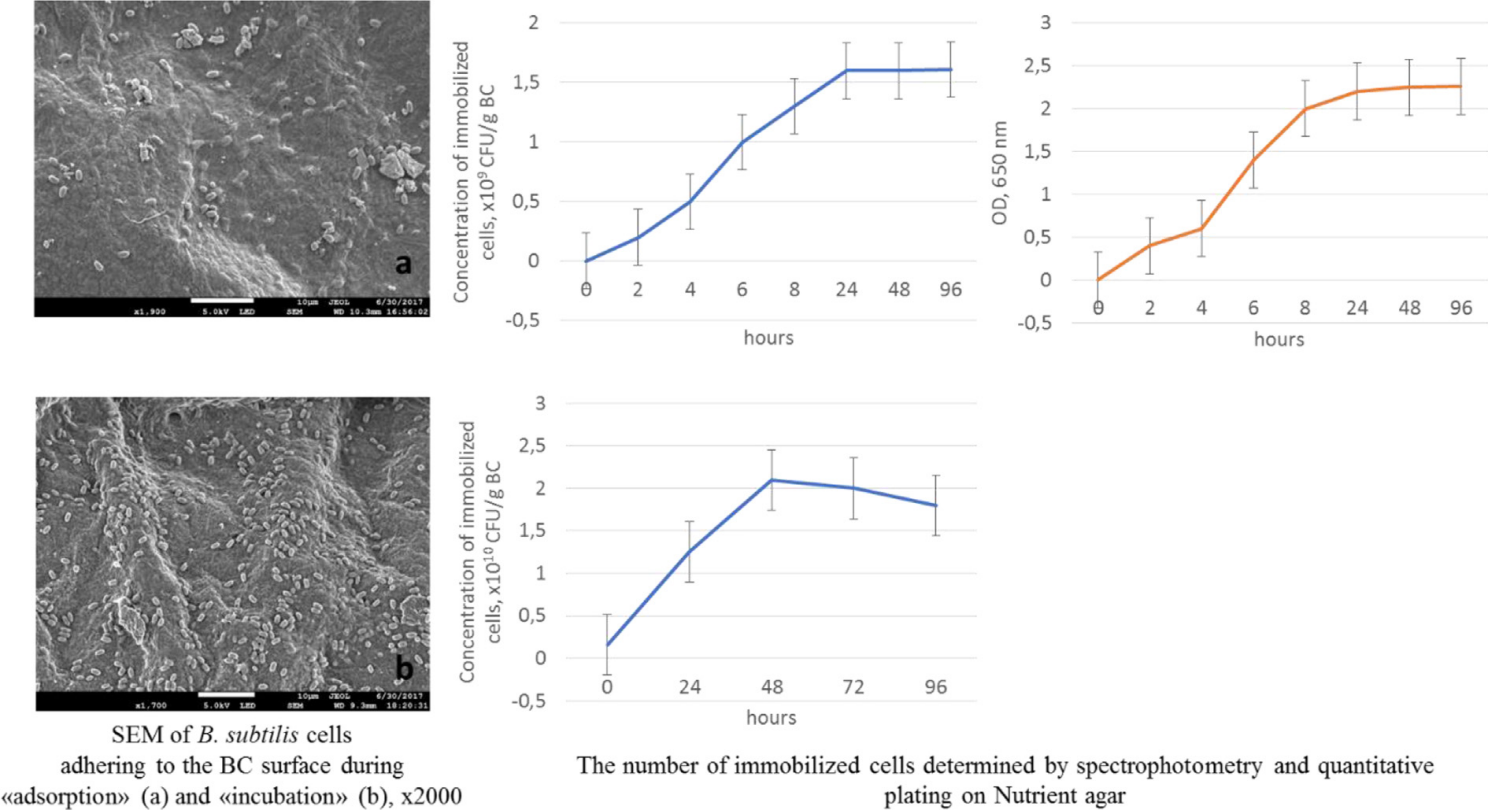
Immobilization efficiency of BS P-2 cells on BC carrier by «adsorption-incubation» method.

A number of bacteria immobilized on it, which was determined from the optical density and quantitative plating of culture suspension on nutrient agar after treatment of films with immobilized cells by cellulase enzyme (Fig. 2), estimated the sorption efficiency of microbial cellulose.

According to the data obtained, 24 h is the optimal time for immobilization of BS P-2 cells on BC gel film on «adsorption step», where cells and films are incubated in a physiological saline. The number of live bacilli in film after 24 h reached 1.6 × 10^9^ CFU/g.

During the adsorption step of the immobilization procedure, bacterial cells adsorbed mostly on the surface of BC pieces. On the «incubation step» the film with immobilized cells was placed in a nutrient broth. BC is spongy enough for nutrition compounds to diffuse into space inside BC so that BS P-2 growth is able to occur. During the incubation step of the immobilization procedure, some bacterial cells moved from the surface into the BC pieces and bacterial growth occurred inside the BC. Therefore, after incubation, the cell immobilization density increased. Increase in incubation time from 0 to 2 days augmented the bacterial cell number in the BC pieces 12.5 times. After 2 day incubation, the bacterial cell number in the biocatalyst reached maximum (2.1 × 10^10^ CFU/g). However, if the incubation time was longer than 2 days, bacterial cell number in the biocatalyst decreased due to the lack of substrates for bacterial metabolism. The similar results were observed by Nguen D.N., et al. as they optimized *Saccharomyces cerevisae* immobilization in bacterial cellulose [45].

SEM results confirmed successful immobilization of BS P-2 cells on the surface of the sorbent. Due to the porous microfibrillar structure of BC film, it can be used as a convenient matrix for BS cells. The immobilization procedure was very simple, easy realizing and inexpensive.

The specific therapeutic and prophylactic effect of preparations based on BS is based on a production of various biologically active substances. Traditionally used probiotic drugs are usually dehydrated, and bacterial cells in them are in anabiotic state. The biologically active substances produced by the microorganisms are inactive in this case and are not able to penetrate into the deep layers of the skin. At the same time gel-like hydrophilic bases provide more favorable conditions for germination of spores, propagation of vegetative cells as well as action of enzymes and a complex of biologically active substances [46, 47]. This serves as a basis for carrying out experimental studies to evaluate the possibility of developing new biocomposite materials for external use on the basis of probiotic antagonists and a modern transdermal base such as BC gel-film. The biological component of these materials is represented by a culture of BS P-2, which is harmless to humans.

**Fig. 3:**
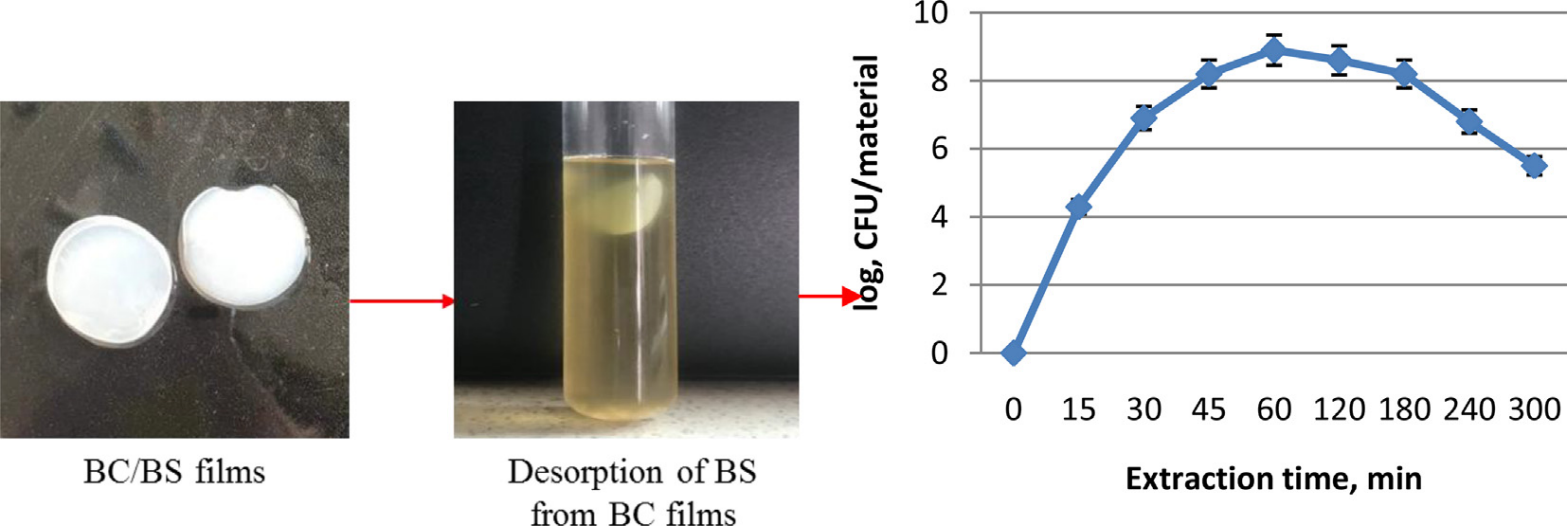
Isolation kinetics of BS P-2 bacteria from bacterial cellulose.

It is expected that upon contact with the wound surface these bacteria will be gradually released. To obtain an information on rate and degree of BS release from the BC/BS biocomposite and to understand better the interactions between the BS and the BC-components, the dynamics of bacteria extraction from gel plates (5 cm^2^) was studied. The samples were placed in 20 ml of PBS and incubated for 5 h at temperature 36 °C in thermostated shaker (Fig. 3). During this time, sampling was carried out and the number of live bacilli was determined by plating successive tenfold dilutions into a potato-glycerin medium.

Already through 15 min, bacterial cells were detected in the medium, and their maximum amount was extracted over a period of 45–120 min, which was log9CFU/gel plate. Diffusion of cells into solution lasted less than 5 h with a gradual decrease in a number of bacteria. Thus, the application of a coating film directly to the wound will allow prolonging an admission of BS into the body.

**Fig. 4:**
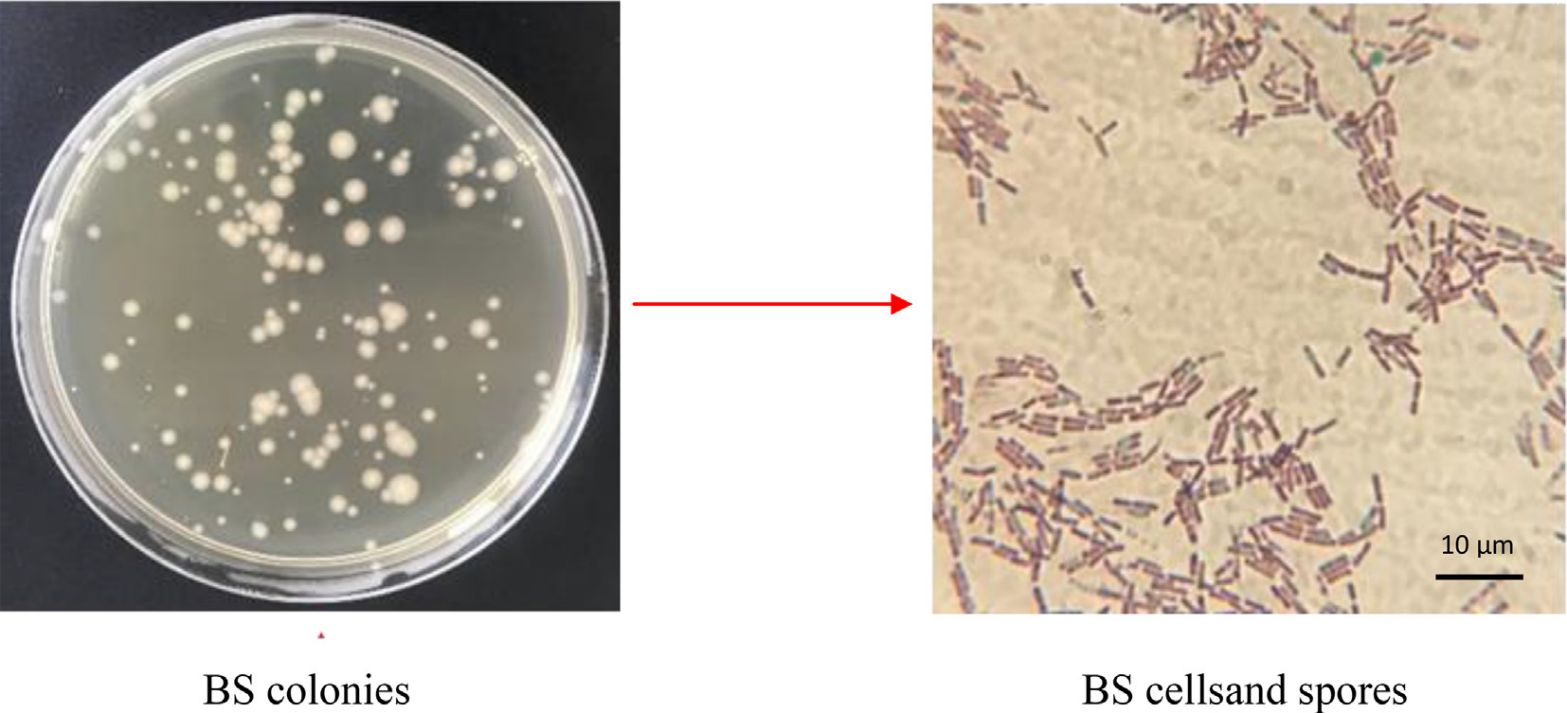
Morphology of colonies and cells of BS P-2 extracted from biocomposites (endospores are stained by Schaeffer Fulton method with Malachite Green dye).

Fig. 4 shows colonies of BS P-2 strain isolated from BC/BS biocomposite.

All colonies are presented in R-forms, about 2 mm in diameter, rough with a wavy edge, opaque, beige or yellowish-beige color, dry consistency. During production of biocomposite, there was no dissociation of microbial population, since all cells formed only R-colonies. Morphological properties of bacteria are similar and characteristic for the first group of *Bacillus* genus of subgroup B: Gram-positive sporeforming rods, size (0.3–0.7) x (1.8–2.2) μm, cells do not swell during sporulation, spores are elliptical, located centrally. These data indicate that the morphological properties of *B. subtilis* P-2 bacteria in the process of this biocomposite production remain stable.

### 3.2 Determination of antagonistic activity of the BC/BS biocomposite

An open wound is a favorable niche for microbial colonization [48]. Antibiotics help in preventing bacterial infection. However, most antibiotics are broadly acting, killing or preventing growth of both good and bad bacteria and opening the door for drug-resistant microbes to cause infection. Despite of several antibiotics being available to treat skin infections, their recurrent use can trigger bacterial resistance https://www.sciencedirect.com/science/article/pii/S0939641117315023 [49]. More than 70% of the bacteria that are responsible for wound infections display resistance to at least one of the antibiotics used in the clinic https://www.sciencedirect.com/science/article/pii/S0939641117315023 [50]. Methicillin-resistant *Staphylococcus aureus* (MRSA) and vancomycin-resistant enterococci are two multi-resistant bacteria that are involved in skin infections https://www.sciencedirect.com/science/article/pii/S0939641117315023 [51]. The number of multidrug resistant bacteria is increasing at an alarming rate, i.e. bacteria are gaining resistance to all known classes of natural and synthetic antibiotics leading to an urgent need for new therapeutic alternatives https://www.sciencedirect.com/science/article/pii/S0939641117315023 [52]. A probiotic bacteria of *Bacillus* genus has been shown to prevent infection while keeping normal skin microbiota in place [27, 53].

Generally, the majority of infected wounds are polymicrobial and are usually contaminated by pathogens found in the surrounding environment, i.e. endogenous microbes living in the mucous membranes, and by the microflora available on the adjacent skin [54]. In the initial stages of chronic wound formation, gram-positive organisms, specifically *S. aureus*, including methicillin resistant (MRSA) are predominant. Furthermore, *Enterococci* species are also found in 50% of chronic wounds. In the later stages, gram-negative *E. coli* and *Pseudomonas* species are observed and tend to invade deeper layers of skin causing significant tissue damage [53]. Therefore, these types of bacteria were used as target microorganisms to determine the antimicrobial activity of BC/BS biocomposite.

The antagonistic activity of BC composite, containing cells of antagonist bacterium, was first determined by agar diffusion test.

**Fig. 5:**
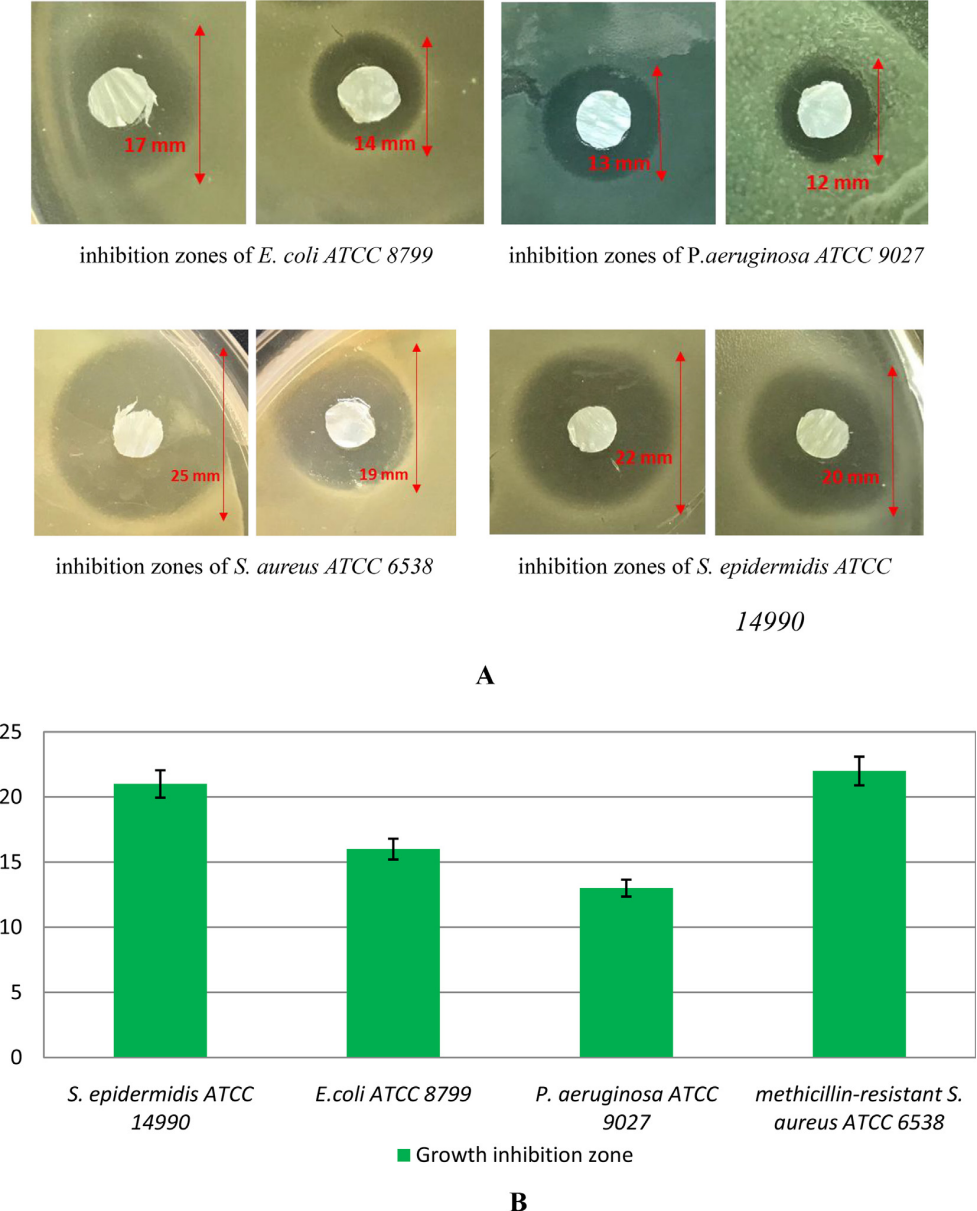
Visualization (A) and average growth inhibition zone sizes (В) of reference bacterial strains during study of an antimicrobial action of BC/BS biocomposite by «agar diffusion» method.

Pieces of pure BC gel film with absorbed cells of *B. subtilis* P-2 were placed on a surface of agar plates previously inoculated by target bacterial suspensions of reference strains of *E. coli* ATCC 8799, *P. aeruginosa* ATCC 9027, methicillin-resistant *S. aureus* ATCC 6538 (MRSA) and *S. epidermidis* ATCC 14990. At the end of incubation, the growth inhibition zones of target microorganisms formed on a matte surface were assessed (Fig. 5).

Here, the binary biocomposite BC/BS was exposed to different reference strains and the microbial activity was investigated. Fig. 5 indicates an antibacterial effect for the biocomposite, evident by the inhibition zone around the fiber mats. The inhibition zone was found to range from 13 to 22 mm. So, the inclusion of BS Р-2 cells into bacterial cellulose leads to an antibacterial efficacy of this biocomposite.

However, since the bacterial growth inhibition does not mean the bacterial death, this method can not distinguish bactericidal and bacteriostatic effects. Moreover, the agar diffusion method is not appropriate to determine the minimum inhibitory concentration (MIC), as it is impossible to quantify the amount of the antimicrobial agent diffused into the agar medium.

Time-kill test is the most appropriate method for determining the bactericidal effect. It is a strong tool for obtaining information about the dynamic interaction between the antimicrobial agent and the microbial strain. The time-kill test reveals a time-dependent or a concentration-dependent antimicrobial effect [55].

**Fig. 6:**
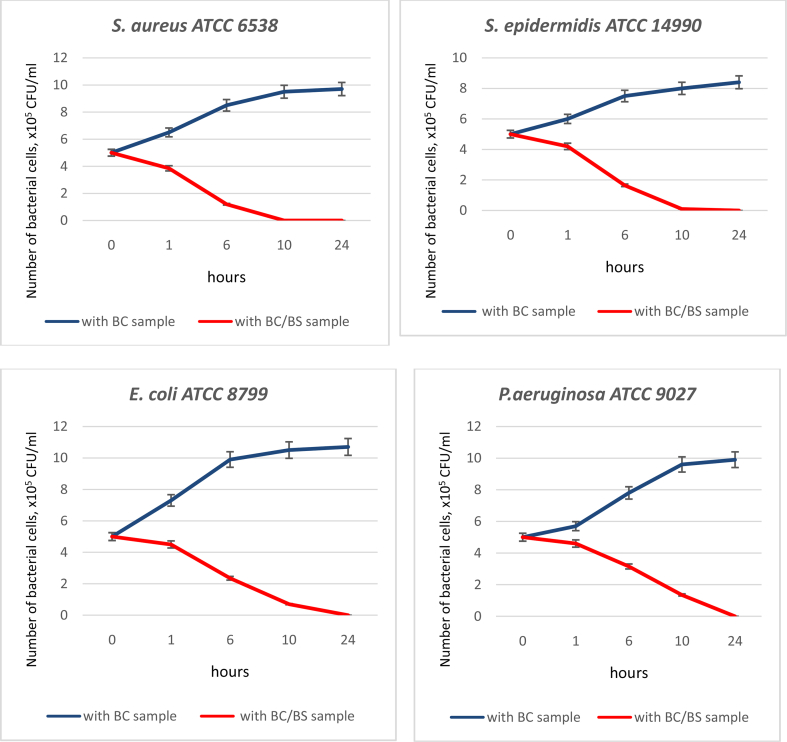
Assessment of antimicrobial activity of BC and BC/BS biocomposite by time-kill test.

The bactericidal activities of the BC and BC/BS biocomposite against the four microorganisms are shown in Table 1 and Fig. 6.

**Table 1:** Reduction ratio of bacterial cells grown with BC/BS biocomposite for the defined period of time (%).

Strains	Time, hours			
	1	6	10	24
*Staphylococcus aureus* ATCC 6538	23 ± 1.1	76 ± 3.8	100	100
*Staphylococcus epidermidis* ATCC 14990	16 ± 0.8	67 ± 3.3	98 ± 1.8	100
*Escherichia coli* ATCC 8799	10 ± 0.5	53 ± 2.6	86 ± 4.3	100
*Pseudomonas aeruginosa* ATCC 9027	8 ± 0.4	37 ± 1.8	73 ± 3.6	100

The BC film did not exert an inhibitory effect. Incubation of target microorganisms with BC films in the broth not only reduced the number of viable bacteria but even made it possible of their reproduction. By the end of the incubation time, the CFU/ml level almost doubled.

Bactericidal activity was indicated by a bacteria growth inhibition rate (%) over time. The normal growth rate of each organism was represented by the growth control, which contained no samples. Overall, the BC/BS biocomposite seemed to be effective against these four tested organisms, especially for the Gram-positive bacteria. The BC/BS biocomposite exerted maximal bactericidal activity, achieving 100% reduction of bacterial growth after 24 h for gram-negative and 10 h for gram-positive bacteria. The killing pattern of *S. aureus* by BC/BS biocomposite was similar to *S. epidermidis.* BC/BS biocomposite exhibited a good bactericidal effect against *E. coli* and *P. aeruginosa*. The maximal killing of *P. aeruginosa* was achieved after 24 h and the reduction in bacterial counts was sustained. Thus, the BC/BS biocomposite had a pronounced bactericidal effect on 4 tested strains.

The obtained results can be explained by the ability of *Bacillus* strains to produce a wide range of antimicrobial compounds [15, 56]. These substances are usually protein- and peptide-based compounds such as enzymes, bacteriocins and lipopeptides [57]. *Bacillus*-derived antimicrobial peptides can be classified according to peptide biosynthesis, structure, and molecular weight. According to their biosynthetic pathway, these metabolites can be grouped into two different classes: the first class comprises ribosomally synthesized peptides, including bacteriocins whereas the second class comprises small microbial peptides synthesized enzymatically by non-ribosomal pathways [16, 17]. The precise mechanism of action of these antimicrobial peptides is not yet clear; however, one proposed mechanism is that these peptides kill bacteria by forming channels in and (or) disrupting the bacterial cell wall. Killing bacteria via the formation of pores in the bacterial membrane requires 3 principal steps: binding to the bacterial membrane, aggregation within the membrane, and formation of channels. Channel formation leads to leakage of internal cell contents and, consequently, cell death [58].

It is possible, that BC/BS biocomposite exhibits sustained release of BS cells resulting in bactericidal action. The material showed its bactericidal activity after the first hour and the activity generally lasted for at least 24 h. One advantage of this antibacterial action is that it allows wound healing to proceed without bacterial interference and reduces the likelihood that resistance will develop. Our study confirmed the effectiveness of BC/BS biocomposite against a standard range of bacterial wound pathogens.

### 3.3 Studying the proteolytic activity of BC/BS biocomposite

The conventional antibacterial agents provide only one aspect of wound treatment – they eliminate bacteria from the wound, thereby preventing invasive infection. An effective wound dressing should also contain an agent that promotes the epithelium regeneration due to splitting of dead tissue. Dead tissue covering the wound also serves as a medium for bacterial growth, reduces the host's resistance to infection, delays the formation of granulation tissue and the re-epithelialization. Proteolytic enzymes (serine proteases, metalloproteinases, cysteine proteases and aspartate proteases) positively contribute to the regeneration processes of tissues. In surgical practice, enzymatic treatment of wounds with proteases produced by BS is already used [20]. Enzymatic debridement with subtilains ointment is widely used in the treatment for pressure sores and leg ulcers [59]. It is assumed that proteases produced by BS support healing of burned skin wounds due to enzymatic debridement effect [60].

**Fig. 7:**
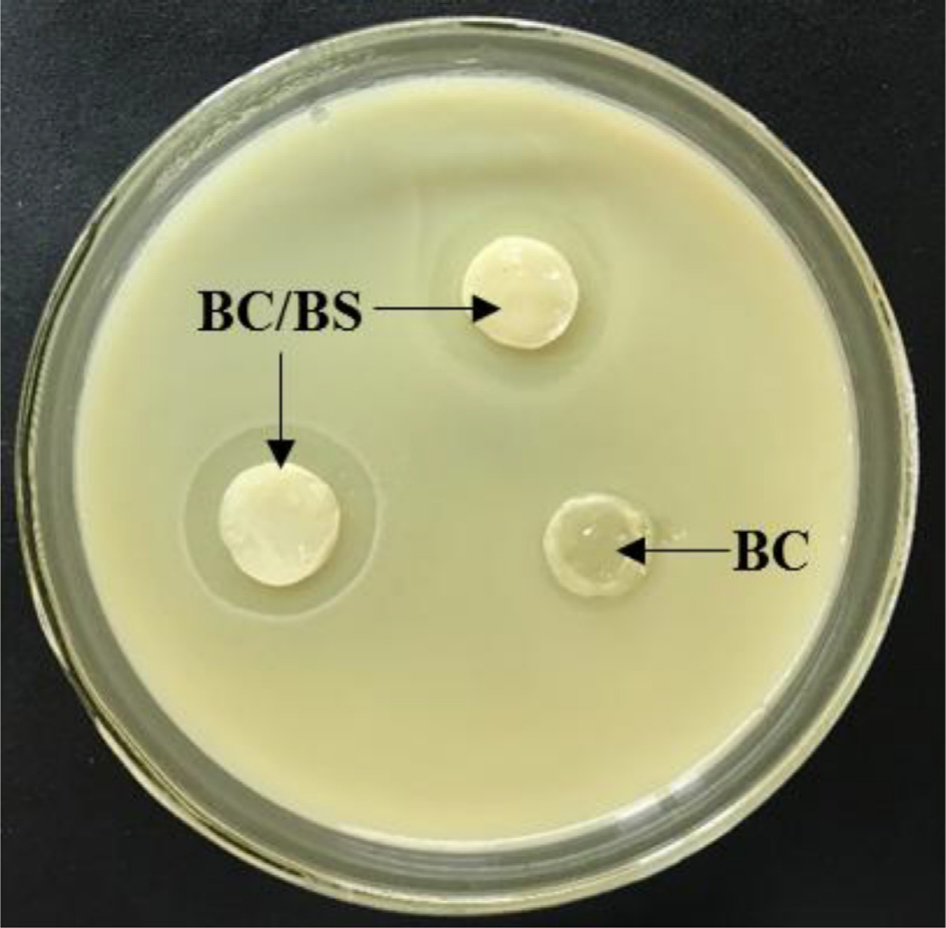
Clear zone on skim milk agar around pure BC film and BC/BS biocomposite.

The BS P-2 strain has a high level of proteases excreted into the culture medium [44, 61]. The release of protease enzymes from BS P-2 incorporated in BC matrix and development of milk-casein digestion zone around the BC/BS biocomposite is shown in Fig. 7.

The appeared clear zones indicated the capability of BS incorporated in BC matrix to produce proteases which digest the milk-casein. The average diameter of the zones was 16 mm. It is supposed that proteolytic enzymes produced by BS cells will be gradually released from such films, subsequently leading to faster wound healing.

### 3.4 Study of wound contraction and epithelialization time when applying the BC/BS biocomposite

The bioactive wound coatings based on the BC with immobilized therein cells of DS destined for the local application. The therapeutic efficacy of the investigated gel-film samples has been evaluated on reducing the wound surface size and the healing time. The results of the action of composite gel films on the healing of excision wounds are presented in Table 2.

**Table 2:** Effect of BC/BS biocomposite and BC films on wound contraction and epithelization period in excision wound.

Group of animals	Wound size, mm					
	0^th^ day	3^rd^ day	5^th^ day	7^th^ day	14^th^ day	21^st^ day
I - BC/BS biocomposite	195 ± 3^a^	83 ± 2^b^	28 ± 5^c^	0	0	0
II - BC film	188 ± 4^a^	105 ± 7^b^	53 ± 2^c^	14 ± 2^c^	0	0
III – Control (without treatment)	192 ± 5^a^	175 ± 4^a^	105 ± 7^a^	64 ± 3^a^	47 ± 2^a^	3.2 ± 0.9^a^

Note: Values are mean ± SEM (Percent) of 5 rats in each group. ^a^*P* < 0.05, ^b^*P* < 0.01, and ^c^*P* < 0.001 compared to respective day control group (statistical analysis was done by one-way analysis of variance followed by Dunnett's test for multiple comparisons).

Based on the data obtained, the greatest wound healing activity has been reached with the biocomposite containing BS cells included in BC film. In this group of rats, the time of complete healing have been 7 days in all animals. Somewhat slower, on day 14, the wound have been healed with the application of the “non-functionalized” cellulose gel film. It is submitted that the wounds are quickly covered with epithelium in a moist environment [1]. BC have a good absorbing capacity and optimal permeability for exudate, which provides the conditions for facilitating wound healing [3, 6, 14]. The use of all experimental variants led to an accelerated recovery of the wound surface in comparison with the control group without treatment (21 days).

**Fig. 8:**
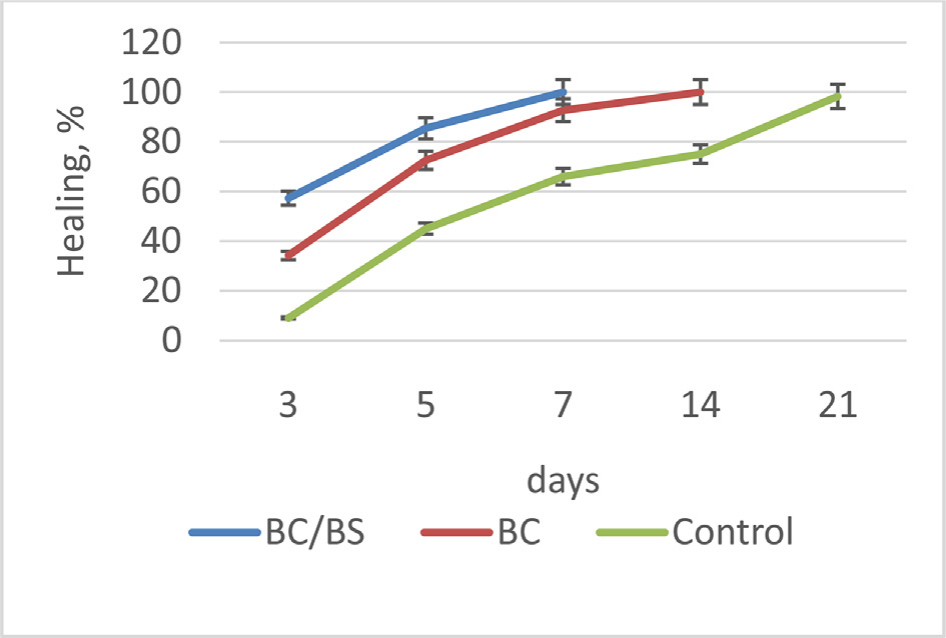
The rate of wound healing in control, group treated with BC/BS (experimental I) and group treated with BC gel-film (experimental II) at different days.

The wound healing ability of BC/BS biocomposite and BC gel-films was also evaluated by monitoring the rate of the wound closure. This rate was evaluated for each group by determination of the closed wound area as a function of time (Fig. 8).

As shown in Fig. 8, significant wound healing activity was observed in animals treated with the BC/BS biocomposite compared with the BC gel-films and control treatment. The percentage of wound closure in the animals treated with BC/BS biocomposite was 57.3% on 3th day; 85.4% on 5th day and 100% on 7th day, respectively. While in experimental group II (BC gel-film), it was 34.2 % on 3th day; 72.5% on 5th day and 92.7% on 7th day, respectively. 100% wound size reduction by BC gel-films was observed on day 14. Thus, the mean time taken for complete epithelialization of the excision wound in BC/BS biocomposite treated group was less than the animals treated with BC gel-films. At the end of the experiment, the untreated rats (physiological solution) still having a small wound (about 1.7%). This finding is in accordance with the literature, in which it has been reported that the natural contraction of wounds takes place by the 21st post wound day [1, 7].

**Fig. 9:**
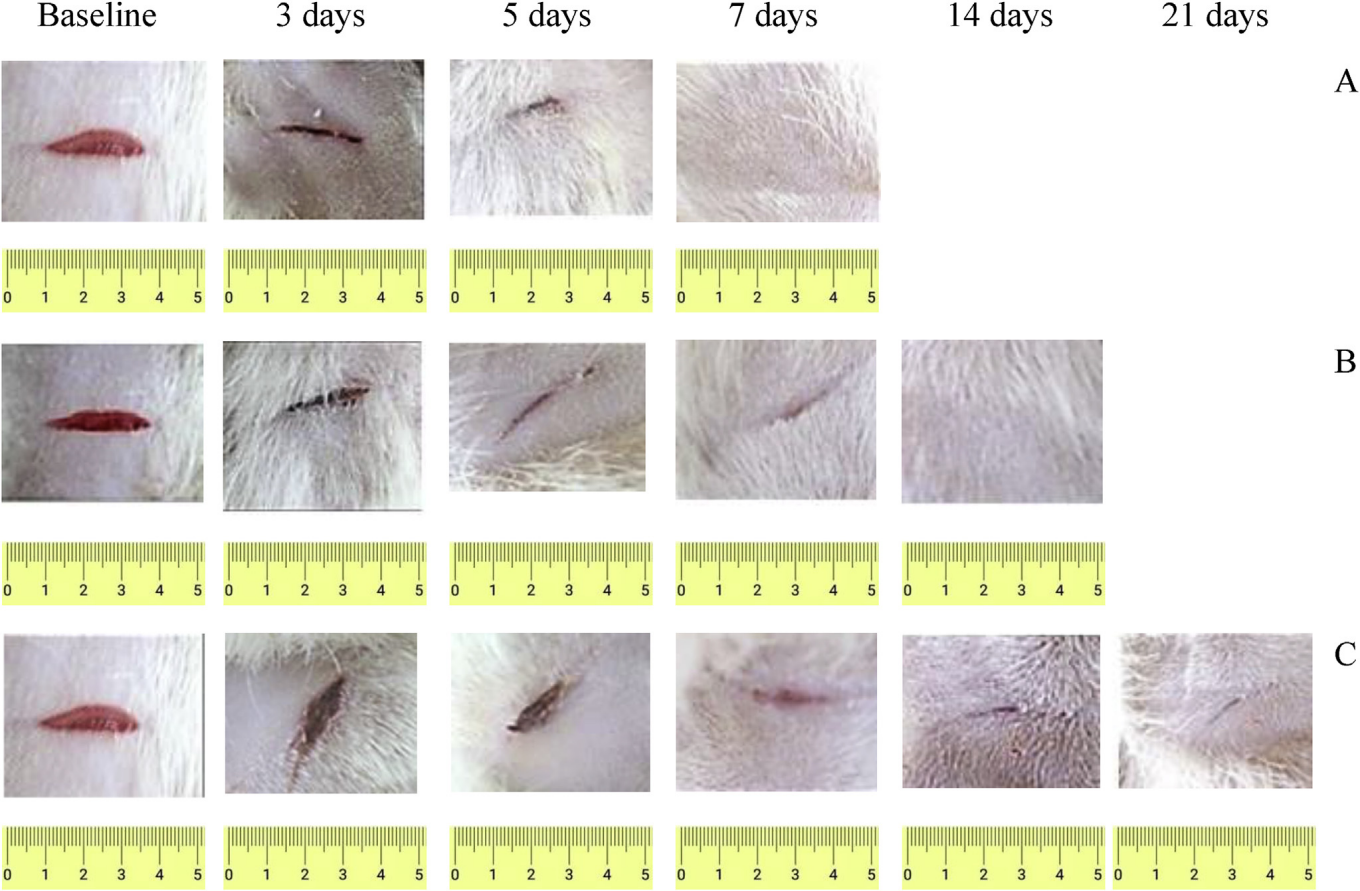
Wound healing effect of experimental samples of coatings based on BC: А – BC/BS; B – BC; С – Control.

Fig. 9 shows the wound photos of all groups.

The bright red coloration observed on the day of induction of wounds reflects the color of the blood that covers the underlying muscle after excision of the skin. The evolution of the wound healing was marked by the brown color that due to the scab formation around the wound. The fall of the scab let to appear a pink color for all groups which reflect the wound reparation and the granulation tissue formation. The visual examination revealed that the wounds were relatively clean and free from any inflammatory reaction like swelling and redness. It should be noted that the replacement of wound dressings did not cause damage to the regenerated wounds, since the films were easily detached from the wound surface. During the wound treatment, bacteria BS P-2 were determined in the amount of 10^4^-10^5^ (the first 7–10 days).

**Fig. 10:**
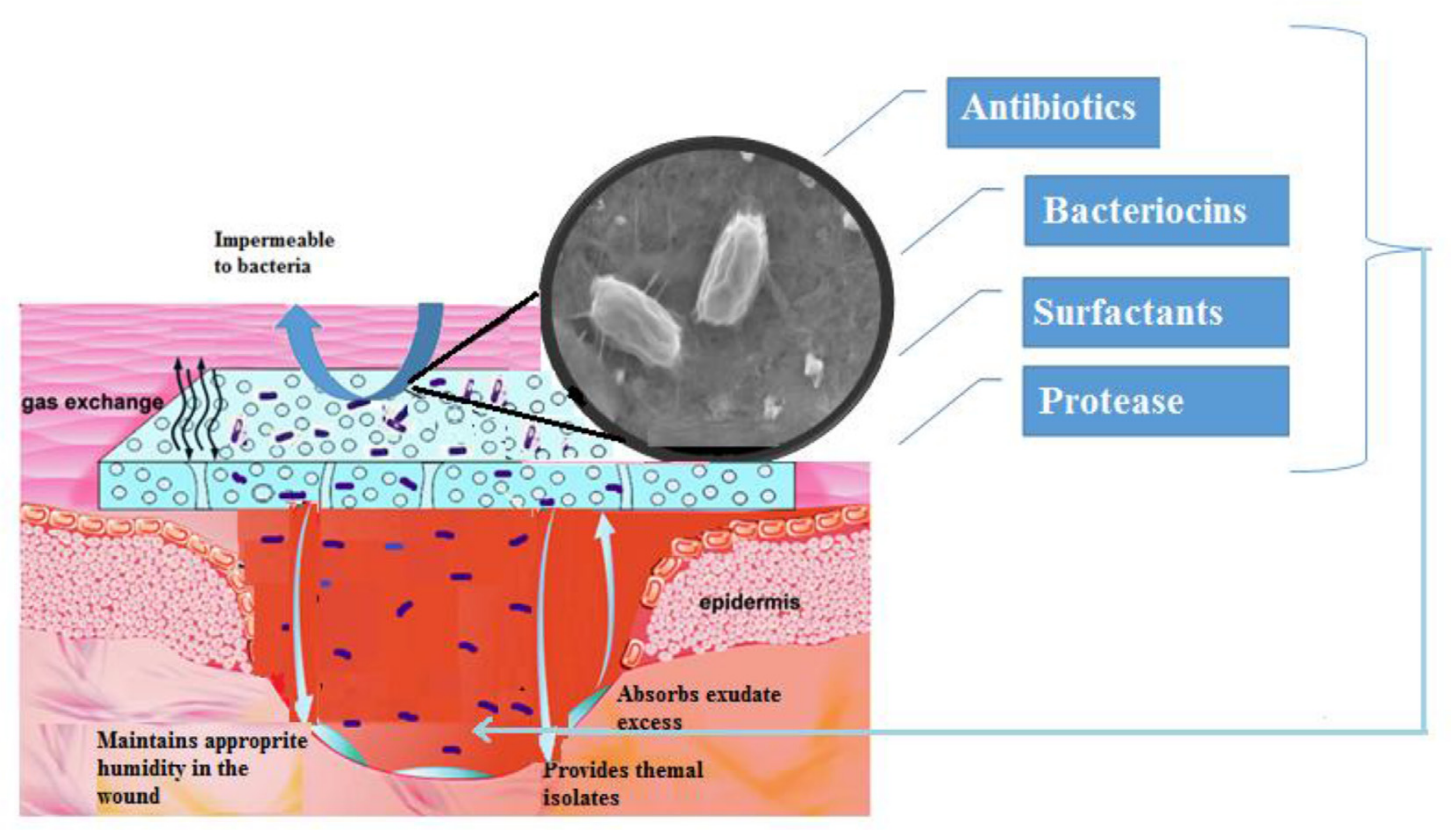
Wound healing mechanisms of BC/BS biocomposite coating.

According to the obtained data in present study, the application of BC/BS biocomposite for wound healing was very advantageous. The current study provides firm evidence supporting the fact that probiotic BS P-2 have positive effect on cutaneous wound healing too. The mechanisms of possible complex therapeutic effect of such a wound dressing are summarized in Fig. 10.

The therapeutic effect is caused by the complex action of functional ingredients included in a biocomposite material. On the one hand, BC gel-film covering the wound provides gas exchange, protects the wound from drying out, prevents the penetration of exogenous infection [2, 5]. In addition, the BC film has pronounced adsorption properties with respect to wound exudate, microbial and tissue degradation products, i.e. contributes to wound cleansing [3, 13]. On the other hand, suppression of infection in the wound, rejection and melting of necrotic tissues are facilitated by BS bacteria diffusing from the cellulose matrix. Living bacterial cells produce antimicrobial substances and proteolytic enzymes that promote the decay of necrotic damaged tissue. BC gel film provides a gradual release of bacteria from it. This allows to prolong the intake of active substances of *B. subtilis* into the body, ensuring their effective “work”. Continuous controlled streaming of protease onto a targeted treated area, provides optimal and controlled environmental conditions for full exploitation of the protease's potential debridement activity. The efficacy of the streaming approach was successfully demonstrated in removal of coagulated blood and debridement of experimental burn wounds in small lab animals by a series of proteases [62]. In addition, the production of proteases by these bacteria facilitates penetration of both the bacteria themselves and metabolites with antibacterial activity produced by them into the wound [19].

The strain used in the work also produces biosurfactants with an antioxidant effect, i.e. can block free radicals [19]. The generation of high level of free radicals during the inflammatory phase may delay wound healing. Indeed, the wound site is rich in both oxygen and nitrogen centered reactive species along with their derivatives. The presence of these radicals will result in oxidative stress leading to lipid peroxidation, DNA damage, and enzyme inactivation, including free radical scavenger enzymes. Therefore, advanced researches were trying to find new and safe bioactive substances with potential antioxidant activity that can accelerate and improve wound healing activity [3]. It was shown that *Bacillus*-produced lipopeptide biosurfactants like surfactin, pumilacidin and fengycin can accelerate and improve wound healing activity [17, 63]. In fact, it has been suggested that the free-radical-scavenging properties of lipopeptides biosynthesized by *B. mojavensis* helped to prevent inflammation and improve the tissue formation, the re-epithelization and differentiation of epidermis [19]. Topical applications of compounds with free-radical-scavenging properties in patients have shown to improve significantly wound healing and protect tissues from oxidative damage. In addition to that, the crude lipopeptide biosurfactant showed a potent inhibition of multidrug resistant bacteria [64]. So, we can suggest that the application of BS lipopeptides based gels is efficient in preventing microbial proliferation in the wound, side causing inflammation and pain.

With the penetration of bacteria, their transient translocation occurs in the underlying layers of skin and in the blood and tissues of the internal environment of the body. When bacteria are destroyed, they serve as a source of antigens to maintain a normal level of antibodies. It is important that DS does not have a damaging effect on tissue; on the contrary, it stimulates the immune processes in the body. It has been demonstrated that administration of BS induces activation of macrophages [65, 66]. In activated macrophages, the synthesis and release of proinflammatory cytokines is enhanced: tumor necrosis factor α, interferon -γ (IFN-γ), interleukin (IL) 1β, IL-6, IL-8, IL-10, IL-12, macrophage inflammatory protein-2. As a result, a complex inflammatory response is developed to destroy the pathogen. A direct ability to stimulate lymphocytes due to cell walls, peptidoglycans and teichoic acids of BS was found [67]. On this basis, it is believed that translocation can be a natural defense mechanism that is appropriate to use in clinical practice, using probiotics not only for prophylaxis and treatment of dysbacteriosis, but also for surgical infection [68, 69].

In the body, vegetative cells turn into spores. Then the spores germinate again in vegetative cells. Germination and resorption cycles are repeated several times until they are removed from the host organism [70]. It was shown that dipicolinic acid present in spores of *Bacillus* spp. probiotic strains inhibits the *in vitro* growth of most pathogenic bacteria [71]. When analyzing the microbial contamination of wounds of various etiologies, it was found that the presence of BS bacteria inhibits the development of purulent inflammation [72]. Consequently, the inhibitory effect of dipicolinic acid in combination with the enhancement of local immunity are new elements of the specific probiosis mechanism of spore probiotics.

Thus, the probiotic effects of spore-forming bacteria can be achieved mainly due to their antagonistic properties: the production of antibiotics and enzymes of others by vegetative cells, the action of dipicolinic acid of spores. Secondly by stimulation of the immunocompetent cells, activation of interferons production. And thirdly, in simultaneous combination of the above-mentioned and other factors (including translocation) that increase the protective response of the organism as a whole. These mechanisms of action make the use of BS as part of complex therapy in composition of wound dressings justified.

## 4 Conclusion

The biocomposite based on BC gel film and BS cells was obtained. Immobilization of BS cells in BC by ‘adsorption- incubation’ method resulted in high cell number in it. The number of antagonist bacteria immobilized on biocomposite was around 10^10^ viable cells per 1 g of the film wet mass. Antibacterial activity of BS is enhanced by the produced lytic enzymes action actively lysing cells of both gram-positive and gram-negative bacteria – the typical causative agents of wound infections.

The BC/BS biocomposite has a high therapeutic efficiency on wound process models. All these factors together can shorten the time needed for treatment of wounds. Overall, these results suggest the promising application of BC/BC biocomposite as a wound dressing, to promote full-thickness wound healing.

## Declarations

### Author contribution statement

Irina Savitskaya: Conceived and designed the experiments; Performed the experiments; Analyzed and interpreted the data; Contributed reagents, materials, analysis tools or data.

Dina Shokatayeva: Performed the experiments.

Aida Kistaubayeva: Conceived and designed the experiments; Wrote the paper.

Ludmila Ignatova: Analyzed and interpreted the data.

Ilya Digel: Contributed reagents, materials, analysis tools or data.

### Funding statement

This work was supported by grant 2679/GF4 «Development of biocomposite materials on the basis of bacterial cellulose for creating the transdermal therapeutic systems» from the Science Committee of the Ministry of Education and Science of the Republic of Kazakhstan.

### Competing interest statement

The authors declare no conflict of interest.

### Additional information

No additional information is available for this paper.
